# Gastrointestinal bleeding during anticoagulation with direct oral anticoagulants compared to vitamin K antagonists

**DOI:** 10.21542/gcsp.2022.21

**Published:** 2022-12-30

**Authors:** Kanchan Dongre, Yasmin Schmid, Luana Nussbaum, Clemens Winterhalder, Stefano Bassetti, Andreas Holbro, Lukas Degen, Anne B. Leuppi-Taegtmeyer

**Affiliations:** 1 Department of Patient Safety, Medical Directorate, University Hospital Basel, Switzerland; 2 Department of Clinical Pharmacology and Toxicology, University Hospital Basel and University of Basel, Basel, Switzerland; 3 Division of Internal Medicine, University Hospital Basel and University of Basel, Basel, Switzerland; 4 Blood Transfusion Centre, Swiss Red Cross, Basel, Switzerland; 5 Department of Hematology, University Hospital Basel and University of Basel, Basel, Switzerland; 6 Clarunis, University Center for Gastrointestinal and Liver Diseases, Basel, Switzerland

## Abstract

**Aim:** Patients receiving oral anticoagulants with vitamin K antagonists (VKAs) and direct oral anticoagulants (DOACs) have an increased risk of gastrointestinal bleeding (GIB). We compared cases of GIB associated with VKAs and DOACs in terms of risk factors, scores, timing, location, severity, and outcome.

**Methods:** Data from patients treated at a university hospital in Switzerland for GIB under VKA and DOACs between 2012 and 2018 were analyzed in this investigator-driven, retrospective, single-center study.

**Results:** A total of 248 patients (110 males; median age, 80 years; 134 VKA, 114 DOAC) were included. No significant differences in age, weight or sex were observed. The propensity of the VKA group for risk factors such as comorbidities, interacting medications, or a high risk for bleeding (HAS-BLED score) was higher than that of the DOAC group. 56% of the VKA-treated patients had a supratherapeutic INR, and 25% in the DOAC group received an unlicensed dose. Significantly more patients in the DOAC group were not formally overdosed with OAC whilst receiving amiodarone compared to the VKA group (57% *vs.* 18%, respectively, *p* = 0.03). Latency between the documented start of oral anticoagulation and GIB was shorter in the DOAC group (median 3 months) than in the VKA group (median 23.5 months, *p*¡0.001). There were no differences in terms of location and severity of the GIB, length of hospitalization, or mortality.

**Conclusion**: Patients taking VKAs displayed more risk factors for GIB than those taking DOACs. Treatment with DOACs was associated with early GIB and calls for increased vigilance during the first months after commencement. Co-medication with amiodarone appeared to be a risk factor for GIB in patients taking DOACs.

## Introduction

Oral anticoagulants (OACs) are used for the prevention and treatment of venous thromboembolism and to reduce the risk of stroke in patients with atrial fibrillation^1,2^. Until 2008, Vitamin K antagonists (VKAs) were the only OACs in use in Switzerland. Since then, they have largely been superseded by direct oral anticoagulants (DOACs)^3^. In Switzerland the licensing of DOACs took place in 2008 (rivaroxaban), 2011 (apixaban), 2012 (dabigatran) and 2015 (edoxaban)^4^.

DOACs are preferable to VKAs in most clinical situations due to their faster onset of action, fewer drug interactions, shorter half-life, wider therapeutic range, and no requirement for routine therapeutic drug monitoring^5^. However, OAC use increases the risk of gastrointestinal bleeding (GIB)^6^.

Previous studies examining the overall bleeding risk of DOACs showed favourable outcomes for DOACs compared with VKAs. A significant reduction in fatal bleeding, intracranial bleeding, clinically relevant non-major bleeding, and total bleeding was observed in patients using DOACs, but no difference was seen in the risk of GIB^7^.

The superiority of DOACs (excluding dabigatran) in terms of GIB risk was observed in a meta-analysis of elderly patients (>75 years)^8,9^. Similarly, a slight increase in GIB risk in patients using dabigatran compared with patients using warfarin was observed in a meta-analysis in 2015^10^. The most recent meta-analysis showed no difference in GIB risk for patients treated with VKAs, rivaroxaban, dabigatran and edoxaban. However, apixaban was associated with a reduced risk for GI bleeding^6^.

The risk of bleeding under anticoagulation is influenced by individual risk factors including polypharmacy or comorbidities^11^, in particular, concomitant use of ulcerogenic drugs (*i.e.,* NSAID, steroids)^12,13^, interacting drugs^14,15^, older age^16^, renal impairment^16^, and a history of GIB^17^.

Nevertheless, differences in risk factors and in manifestations of GIB associated with the use of DOACs, compared to VKAs, are not yet fully elucidated. We therefore performed a retrospective analysis of cases of GIB under DOACs and GIB under VKAs with the aim to compare risk factors, timing, severity, and outcome.

## Materials and methods

### Study design

The study was designed as an investigator-driven, single-centre, retrospective study of cases of GIBs associated with DOACs or VKAs at the University Hospital Basel (USB) between January 2012 and January 2018. The study was approved by the ethics committee “Nordwest- und Zentralschweiz” (EKNZ, project-ID: 2018-00066) and was conducted in full compliance with national ethical and regulatory guidelines.

**Figure 1.**
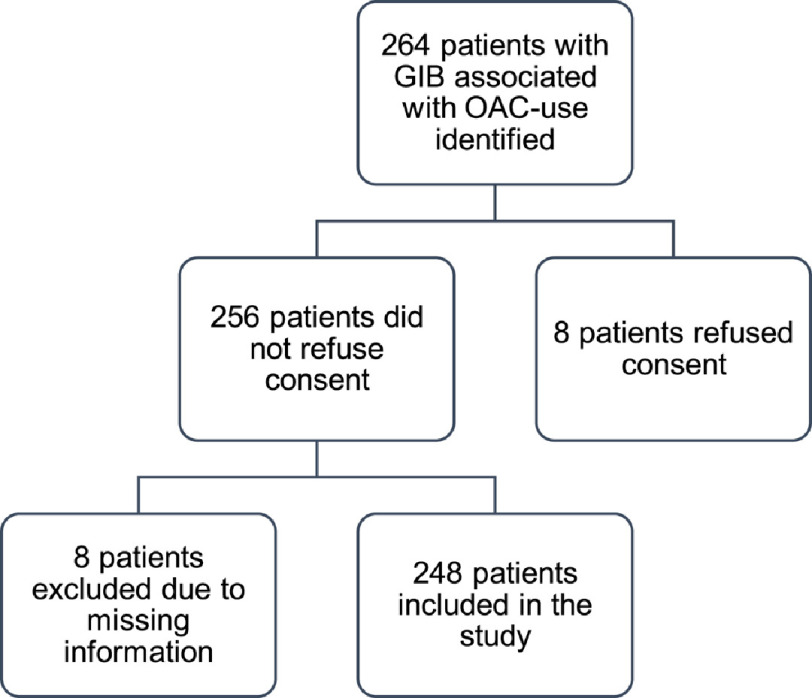
Study flow chart.

### Study population

Patients who had experienced a GIB under treatment with an OAC were identified from the Department of Clinical Pharmacology‘s “Adverse drug reactions ward round” database. Adult patients (18 years or older) who experienced a GIB associated with OAC immediately prior to or during hospitalization in the Department of Internal Medicine, USB, between January 2012 and January 2018 were included in the study. Patients who experienced re-bleeding under OAC were recorded as two separate cases. All patients consented to the analysis of their data. Figure 1 shows the study flow chart.

### Data sources

Data was retrieved from the hospital’s electronic medical information system and electronic drug-prescription charts as well as from paper records. The number of blood transfusions, platelet transfusions and fresh frozen plasma administrations during the hospitalization was provided by the local blood transfusion centre.

**Table 1:** Patient characteristics. Asterisks (*) represent the *p*-values derived from chi-square tests. *P*-values indicating significant differences are given in bold and italics. HAS-BLED and CHA2DS2-VASc scores were only calculated for patients with atrial fibrillation.

	Entire study population	DOAC	VKA	*P*-Value
Number (%)	248	114 (46)	134 (54)	
**Patient characteristics**				
Male, n (%)	110 (44)	54 (47)	56 (42)	0.450*
Age (years), median (IQR)	80 (14)	80 (13.75)	80.5 (14.75)	0.909
Weight (kg), mean ± SD	72.9 ± 18.3 (*n* = 240)	73.7 ± 20.7 (*n* = 111)	72.3 ± 16.0 (*n* = 129)	0.553
BMI (kg/m^2^), mean ± SD	26.2 ± 5.6 (*n* = 226)	26.4 ± 6.4 (*n* = 104)	25.1 ± 4.8 (*n* = 122)	0.402
Height (cm), mean ± SD	166.6 ± 14.7 (*n* = 226)	167.0 ± 10.1 (*n* = 104)	166.4 ± 9.4 (*n* = 122)	0.68
**Smoking status, n (%)**				
Current	34 (14)	14 (12)	20 (15)	0.06*
Previous	83 (33)	30 (26)	53 (40)	
Never	79 (32)	43 (38)	36 (27)	
Unknown	52 (21)	27 (24)	25 (19)	
**Alcohol consumption, n (%)**				
>8 drinks/week	31 (12.5)	13 (11.4)	18 (13.4)	0.863*
≤8 drinks/week	149 (60.1)	68 (59.6)	81 (60.4)	
Unknown	68 (27.4)	33 (29.9)	35 (26.1)	
**Indication for OAC, n (%)**				
nvAF	156 (62.9)	80 (70.2)	76 (56.7)	<0.0001*
DVT prophylaxis	12 (4.8)	11 (9.6)	1 (0.7)	
DVT treatment	6 (2.4)	1 (0.9)	5 (3.7)	
PE treatment	7 (2.8)	7 (6.1)	0 (0.0)	
Secondary Prevention for DVT/PE	37 (14.9)	12 (10.5)	25 (18.7)	
Heart valve replacement	17 (6.9)	1 (0.9)	16 (11.9)	
Others	13 (5.2)	2 (1.8)	11 (8.2)	
**Charlson comorbidity index**				
mean ± SD	7.0 ± 2.4	6.5 ± 2.1	7.5 ± 2.6	***0.003***
**HAS-BLED score**				
mean ± SD	3.6 ± 1.2 (*n* = 156 )	3.4 ± 1.1 (*n* = 80)	3.9 ± 1.2 (*n* = 76)	***0.005***
**CHA**_**2**_ **DS**_**2**_ **-VASc score**				
mean ± SD	4.3 ± 1.6 (*n* = 156)	4.2 ± 1.6 (*n* = 80)	4.3 ± 1.2 (*n* = 76)	0.576
**Drug prescriptions, n (%)**				
Numbers of drugs used daily, mean ± SD	8.3 ± 3.2	8.2 ± 3.2	8.4 ± 3.2	0.53
<5 drugs/day	30 (12.1)	15 (13.3)	15 (11.2)	0.764*
≥5 drugs/day	217 (87.9)	98 (86.7)	119 (88.8)	
PPI or antacids prior to hospitalization	152 (61.5) (*n* = 247)	73 (64.6) (*n* = 113)	79 (59.0) (*n* = 134)	0.439*
**Prescribed OAC**				
Phenprocoumon			131 (97.8)	
Acenocoumarol			1 (0.7)	
Warfarin			2 (1.5)	
Rivaroxaban		80 (70.2)		
Apixaban		24 (21.1)		
Edoxaban		1 (0.9)		
Dabigatran		9 (7.9)		

**Notes.**

DOAC direct oral anticoagulant VKA vitamin K antagonist IQR interquartile range SD standard deviation BMI body mass index OAC oral anticoagulation nvAF non-valvular atrial fibrillation DVT deep vein thrombosis PE pulmonary embolism PPI proton pump inhibitor

### Outcome measures

The primary outcomes of interest were risk factors associated with GIB and the severity of GIB under DOACs and VKAs. Data recorded included demographic information, laboratory tests, comorbidities, prescription details (including dose and start and stop dates), co-medication and lifestyle variables such as body mass index (BMI), smoking, and alcohol consumption. Polypharmacy was defined as the intake of 5 or more different drugs per day. Co-medication was recorded according to the classification list given in Supplemental Table 1.

In order to systematically assess comorbidities, patients’ predicted 10-year mortality and individual bleeding risk, the Charlson Comorbidity Index (CCI)^18^ and HAS-BLED scores^19,20^ at admission were recorded. GIB was classified as “upper GIB”, “lower GIB” or “unknown” according to the endoscopic findings and description of the origin of bleeding in the patient records.

The Glasgow-Blatchford Bleeding Score (GBS)^21^ and the Rockall score^22^ were calculated to determine the severity and risk of rebleeding of upper GIB^23^, whereas the CURE Hemostasis prognosis score^24^ was calculated to determine the severity of lower GIB. Patient outcome was assessed in terms of survival and length of hospital stay.

## Statistical analysis

A descriptive statistical analysis was performed using Microsoft Excel (Microsoft Corporation 2016). Patient characteristics for each group (DOAC or VKA) were described as proportions, means (±  standard deviation, SD), or medians (interquartile ranges). If the exact day of the start of OAC was unknown, it was imputed to be the first day of the given month. If the exact month was missing, the beginning of OAC was assigned to be 1st January of the recorded year. To test for differences between the groups (DOAC *vs.* VKA), two-tailed student’s t-tests were performed for assessing differences in means of numerical data, and a Chi-square test for comparing the distribution of categorical variables. A *p*-value of <0.05 was considered to be statistically significant. Statistical analyses were performed using Excel and “VassarStats: Website for Statistical Computation”^25^.

## Results

### Patient characteristics

**Figure 2.**
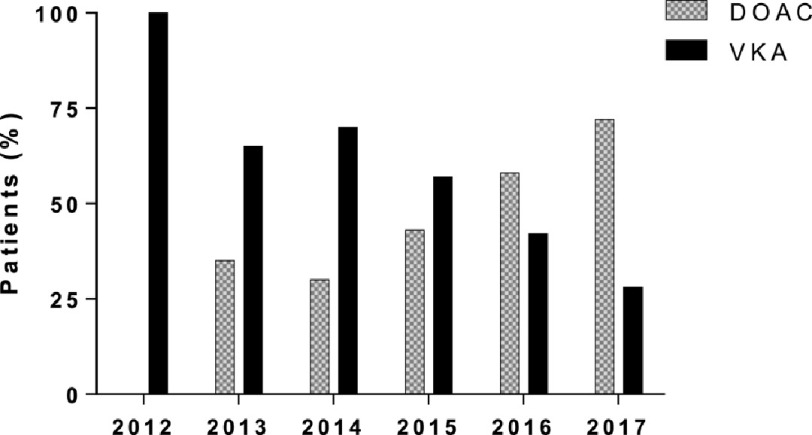
Proportion of prescriptions of direct oral anticoagulants (DOAC) and vitamin K antagonists (VKA) in patients presenting at the University Hospital Basel with gastrointestinal bleeding (GIB) over the years.

Patient characteristics are shown in Table 1. Among 248 identified patients (110 men, 138 women) experiencing a GIB under OAC, 114 (46%) were taking a DOAC (rivaroxaban *n* = 80, apixaban *n* = 24, dabigatran *n* = 9, edoxaban *n* = 1), and 134 (54%) were taking VKAs (phenprocoumon *n* = 131, warfarin *n* = 2, acenocoumarol *n* = 1). Figure 2 shows the distribution of DOAC and VKA use during the study period. Over time, more patients were prescribed a DOAC (from 0% in 2012 to 72% in 2017). No significant differences in age (median age 80 years in both groups), weight, height or sex were observed between the two groups. OAC was mostly prescribed for non-valvular atrial fibrillation (nvAF) with similar CHA_2_DS_2_-VASc scores in both groups at admission (Table 1). Patients taking VKAs had significantly higher Charlson comorbidity indexes and HAS-BLED scores at admission (Table 1).

**Figure 3.**
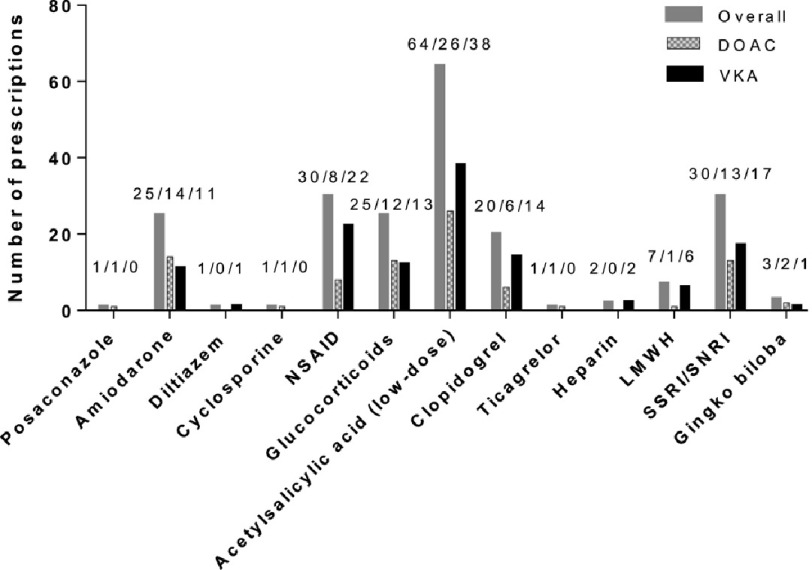
Numbers of prescriptions of different possibly interacting drug groups or active substances in patients presenting with gastrointestinal bleeding under oral anticoagulation. From left to right: the first four drugs interact pharmacokinetically with OAC while the remaining drugs all primarily interact pharmacodynamically. DOAC, direct oral anticoagulant; VKA, vitamin K antagonist; NSAID, non-steroidal anti-inflammatory drug; LMWH, low-molecular-weight heparin; SSRI, selective serotonin reuptake inhibitor; SNRI, selective noradrenaline reuptake inhibitor.

Polypharmacy was common in both groups (>86%) (Table 1). Overall, 139 (56.3%) patients received at least one drug with interaction potential with the OAC. Pharmacodynamic interactions were more common than pharmacokinetic interactions. Figure 3 shows the distribution of prescriptions among different interacting drug groups or active substances. Patients taking DOACs were less likely to have concomitant interacting drugs than patients taking VKAs (47.8% *vs* 63.4%, had one or more potentially interacting drug, *p* = 0.033). The frequency of possible pharmacokinetic interactions did not differ between groups.

In contrast, pharmacodynamic interactions were borderline significantly more common in the VKA group than in the DOAC group (59% of the VKA group had at least one pharmacodynamic interaction *vs.* 43% of the DOAC group; *p* = 0.05). The most frequently observed possibly interacting drug among the pharmacokinetic interactions was amiodarone (10% of all patients). Regarding the pharmacodynamic interactions, low-dose acetylsalicylic acid was the most commonly prescribed drug (overall *n* = 64, 25.9%), followed by NSAIDs (overall *n* = 30, 12.1%) and glucocorticoids (overall *n* = 25, 9.1%). In both groups, more than half of the patients were already prescribed a proton pump inhibitor or antacids at admission (64.6% in the DOAC group, and 59.0% in the VKA group, respectively; *p* = 0.439).

At admission, mean INR was 4.5 (SD 3.1) and 56% of VKA-treated patients had a supratherapeutic INR (Table 2). Twenty-eight (25%) of all patients in the DOAC group received an anticoagulant dose which was different from the licensed dose (the commonest cause for discrepancy was impaired renal function). Five patients were prescribed a DOAC when it was formally contraindicated (4.5% of the DOAC group). Eighteen patients received a DOAC at too high a dose (16% of the DOAC group) and 10 patients were prescribed a DOAC at a too low dose (9% of the DOAC group) according to their renal function and with respect to their age, weight, and indication for the OAC (Table 2).

**Table 2:** Anticoagulation characteristics. Numbers are number of patients (%) of all patients treated with drug for whom complete information was available to assess dose correctness (eGFR, age, weight, interacting medication).

	Admission INR in target range	Admission INR above target range	Admission INR below target range
Vitamin K antagonist(*n* = 132)	35 (26.5)	75 (56.0)	22 (17.5)
**DOAC**	**Recommended dose**	**Exceeds recommended dose**	**Less than recommended dose**
Rivaroxaban (*n* = 80)	60 (75)	15 (19)	5 (6)
Apixaban (*n* = 22)	18 (82)	1 (4.5)	3 (13.5)
Dabigatran (*n* = 8)	4 (50)	2 (25)	2 (25)
Total DOAKs (*n* = 110)	82 (75)	18 (16)	10 (9)

**Notes.**

INR international normalized ratio eGFR estimated glomerular filtration rate

We went on to perform a subgroup analysis of patients receiving amiodarone and OACs, because being a CYP3A4 and P-glycoprotein inhibitor, amiodarone has the potential to interact with all VKAs and all DOACs. Of the 11 patients receiving VKAs in combination with amiodarone, 9 (82%) had a supratherapeutic INR at admission. This compares to 66 (55%) of VKA-treated patients not receiving amiodarone who had a supratherapeutic INR at admission. Among patients receiving a DOAC in combination with amiodarone, 5 (36%) patients were overdosed, 6 (43%) were correctly dosed and 3 (21%) were underdosed. For patients receiving DOACs without amiodarone, these figures were 13 (14%), 76 (79%), and 7 (7%), respectively ( *p* = 0.014 compared to patients receiving amiodarone). Significantly more patients in the DOAC group were not formally overdosed whilst receiving amiodarone compared to the VKA group (57% *vs.* 18%, respectively, Fisher exact probability *p* = 0.03).

### Characterization of the bleeding and clinical presentation at admission

Bleeding characteristics, including latency between starting OAC and experiencing GIB, severity scores, clinical presentation, and laboratory values at admission are shown in Table 3. A significant difference in latency between commencing the OAC and the GIB was observed between the groups, with GIB occurring within a few months of starting a DOAC compared to some years after starting a VKA.

**Table 3:** Bleeding characteristics at admission including severity scores, clinical presentation, and laboratory values. Data represent mean ± standard deviation (SD), unless otherwise stated. Asterisks (*) represent chi-square tests. ‡ indicate comparison of all three groups, # indicate comparison of upper and lower GIB. Numbers in parentheses represent available cases of the corresponding variable, unless otherwise stated. Reference values are listed next to the respective laboratory values.

	Entire study population (*n* = 248)	DOAC (*n* = 114)	VKA (*n* = 134)	*P*-Value
**Latency time between starting OAC and GIB**				
Time (months), median	28.5 ± 53, 196	6.5 ± 8.96, 3	47.13 ± 65.55, 23.5	***<0.001***
**Localization of GIB, n (%)**				
Upper GIB	126 (50.8)	50 (43.9)	76 (56.7)	0.117 * ‡
Lower GIB	70 (28.2)	38 (33.3)	32 (23.9)	0.070 #
Unknown localization	52 (21.0)	26 (22.8)	26 (19.4)	
**Scores for upper GIB**				
Glasgow-Blatchford Bleeding score	11.5 ± 4.0 (*n* = 117)	10.8 ± 4.3 (*n* = 49)	12.0 ± 3.7 (*n* = 68)	0.118
Rockall score	6.1 ± 1.7 (*n* = 118)	6.0 ± 1.8 (*n* = 49)	6.2 ± 1.5 (*n* = 69)	0.703
**Score for lower GIB**				
CURE Hemostasis prognosis score	2.7 ± 0.9 (*n* = 69)	2.7 ± 1.0 (*n* = 38)	2.7 ± 0.9 (*n* = 31)	0.884
**Clinical presentation at admission, n (%)**				
Systolic blood pressure	*n* = 237	*n* = 112	*n* = 125	
<100 mmHg	43 (18.1)	23 (20.5)	20 (16.0)	0.462*
≥100 mmHg	194 (81.9)	89 (79.5)	105 (84.0)	
Heart rate	*n* = 236	*n* = 112	*n* = 124	
<100 bpm	184 (78)	90 (80.4)	94 (75.8)	0.493*
≥100 bpm	52 (22)	22 (19.6)	30 (24.2)	
**Laboratory values at admission**				
Hemoglobin (140 –180 g/l)	84.8 ± 27.6	82.7 ± 27.1	86.6 ± 27.8	0.271
Platelet count (150–450 x10^9^/l)	269.6 ± 117.2	261.3 ± 112.2	276.7 ± 120.9	0.305
Leucocytes (3.50–10.00 ×10^9^/l)	9.1 ± 3.9	8.7 ± 3.4	9.5 ± 4.2	0.104
Neutrophils (1.300–6.700 ×10^9^/l) median [IQR]	6.3 [3.9] (*n* = 242)	6.0 [3.8] (*n* = 109)	6.9 [3.8] (*n* = 133)	***0.030***
CRP (<10.0 mg/l)	24.2 ± 43.5 (*n* = 241)	25.6 ± 49.2 (*n* = 110)	23.1 ± 38.1 (*n* = 131)	0.650
Procalcitonin (<0.1 ng/ml)	4.5 ± 14.4 (*n* = 17)	8.9 ± 21.3 (*n* = 7)	1.4 ± 3.5 (*n* = 10)	0.321
Fibrinogen (1.7–4.0 g/l)	5.1 ± 15.0 (*n* = 69)	3.2 ± 1.4 (*n* = 29)	6.5 ± 19.5 (*n* = 40)	0.367
INR (<1.3), median [IQR]		n.a.	2.4 [3.1] (*n* = 133)	
aPTT (25 –34 s)	38.0 ± 15.6 (*n* = 69)	37.8 ± 18.0 (*n* = 28)	38.1 ± 13.7 (*n* = 41)	0.936
Thrombin time (16 –25 s)	27.7 ± 28. 8 (*n* = 71)	32.2 ± 36.0 (*n* = 28)	24.8 ± 22.4 (*n* = 43)	0.296
Factor V (%)	90.2 ± 25.6 (*n* = 60)	76.8 ± 28.3 (*n* = 24)	99.1 ± 18.8 (*n* = 36)	***<0.001***
Factor II (%)	46.3 ± 29.6 (*n* = 60)	64.7 ± 23.9 (*n* = 24)	34.1 ± 26.6 (*n* = 36)	***<0.001***
Factor VII (%)	42.4 ± 32.8 (*n* = 60)	67.6 ± 27.7 (*n* = 24)	25.6 ± 23.9 (*n* = 36)	***<0.001***
Rivaroxaban-level (ng/ml)		170.9 ± 147.8 (*n* = 17)	n.a.	
eGFR (>90 ml/min/1.7 m^2^ CKD-EPI)	50 ± 24 (*n* = 235)	53 ± 24 (*n* = 111)	48 ± 23 (*n* = 124)	0.096
Serum urea (3.4 –8.7 mmol/l)	16.4 ± 12.3 (*n* = 243)	15.3 ± 13.0 (*n* = 111)	17.3 ± 11.7 (*n* = 132)	0.201

**Notes.**

DOAC direct oral anticoagulant VKA vitamin K antagonist GIB Gastrointestinal bleeding bpm beats per minute IQR interquartile range CRP C-reactive protein INR international normalized ratio aPTT activated partial thromboplastin time sec seconds n.a. not applicable eGFR estimated glomerular filtration rate

Of all the patients studied, 126 (51%) were diagnosed with an upper GIB and 70 (28%) with a lower GIB. For 52 (21%) patients, the location of bleeding could not be determined with accuracy because there were no clear stigmata of recent hemorrhage on endoscopy, or endoscopy could not be performed, or the patients’ symptoms were attributable to either an upper or a lower GIB. There was a trend towards upper GIB being more common among VKA users than among DOAC users, and lower GIB was more common among DOAC users than among VKA users (Table 3).

No differences in GBS or Rockall scores were observed when determining the severity of upper GIB using DOACs and VKAs (Table 3). Overall, most patients experiencing an upper GIB under DOACs or VKAs were at moderate risk of death (Rockall score 4-7, 69.5%), and 24.6% were at high risk of death (Rockall score ≥ 8) with no difference between groups. Similarly, when determining the severity of lower GIB, no difference in the CURE hemostasis prognosis score was observed between patients receiving DOACs and VKAs (Table 3). Most patients (84.1%) had a score of 3 or below.

At admission, most patients were hemodynamically stable, and there were no significant differences between the DOAC and VKA users in terms of heart rate or blood pressure (Table 3). A combination of a systolic blood pressure of <100 mmHg and an increased heart rate of >100 bpm was observed in 3.2% of all patients (3.5% in the DOAC group and 3.0% in the VKA group). However, 149 (60%) patients were taking beta-blockers at the time of admission.

**Table 4:** Use of blood products, clotting factors, antagonists and proton pump inhibitors in the management of GIB. Numbers are numbers of patients (%) unless otherwise stated. Asterisks (*) represent chi-square tests. Significant differences are marked in bold and italics. Numbers in parentheses represent available cases of the corresponding variable, unless otherwise stated.

	Entire study population (*n* = 248)	DOAC (*n* = 114)	VKA (*n* = 134)	*P*-Value
**Blood products**				
Erythrocyte transfusions	180 (72.6)	80 (70.2)	100 (74.6)	0.522*
Platelet transfusions	3 (1.2)	2 (1.8)	1 (0.7)	0.888*
Fresh frozen plasma	14 (5.6)	6 (5.3)	8 (6.0)	1.000*
**Pharmacological interventions per patient, mean ± SD**	2.15 ± 1.34 (*n* = 240)	1.63 ± 1.13 (*n* = 113)	2.62 ± 1.34 (*n* = 127)	***<0.001***
**Antagonists**				
All antagonists	128 (53.3)	23 (20.4))	105 (82.7)	***<0.0001*** *
Vitamin K	127 (52.9)	22 (19.5)	105 (82.7)	***<0.0001*** *
Idarucizumab	2 (0.8)	2 (1.8)	0 (0.0)	0.502*
**Coagulation Factor concentrates**				
All coagulation factor concentrates	50 (20.9)	14 (12.4)	36 (28.6)	0.004*
**Prothrombin complex concentrates**				
Factor II, VII, IX, X (Prothromplex^®^) Factor II, VII, VIX, X, Protein C, Protein S, Antithrombin III (Beriplex^®^)	48 (20.1)	13 (11.5)	35 (27.8)	0.003*
**Single factor concentrates**				
Factor VIIa (NovoSeven^®^)	2 (0.84)	1 (0.88)	1 (0.79)	0.527*
**Fibrinogen concentrates**				
Fibrinogen (Haemocomplettan^®^)	1 (0.42)	0 (0.0)	1 (0.79)	1.000*
**Antifibrinolytic agents**				
Tranexamic acid	20 (8.4)	10 (8.8)	10 (7.9)	1.000*
**Other pharmacological treatments**				
Proton pump inhibitors	231 (96.3)	107 (94.7)	124 (97.6)	0.389*

**Notes.**

DOAC direct oral anticoagulant VKA vitamin K antagonist SD standard deviation

At admission, most patients (86.3%, *n* = 214) had hemoglobin (Hb) levels <120 g/l. However, the mean Hb levels at admission did not differ between patients taking DOACs and those receiving VKAs. The range of Hb levels was 33-155 g/l in the DOAC, and 21-179 g/l in the VKA group. Platelet counts also did not differ between the DOAC and VKA groups. Overall, 35 patients (14%, *n* = 18 in the DOAC group and *n* = 17 in the VKA group) had platelet counts below the lower limit of normal (150 ×10^9^/l). Factors II, V, and VII were significantly reduced in patients receiving VKAs compared to those receiving DOACs (all *p*¡0.001). One hundred twenty-six patients (53.6%) had impaired renal function with estimated glomerular filtration rates (eGFR, CKD-EPI) below 49 ml/min/1.7m^2^ (46.5% of the patients in the DOAC, and 54.5% of the patients in the VKA group, respectively). Increased serum urea levels were observed in most patients (69.1%), and no difference between the groups was observed (Table 3).

### Medical management

A comparison of the medical management of GIB between the two groups is shown in Table 4. Significantly more pharmacological interventions were performed in patients experiencing a GIB under VKAs compared with those under DOACs (2.62 ± 1.34, and 1.63 ± 1.13 interventions per patient, respectively; Table 4). No differences were observed in the number of red blood cells or platelet transfusions, administration of fresh frozen plasma, fibrinogen concentrates, antifibrinolytic agents (tranexamic acid), or proton pump inhibitors. Vasopressors, including adrenaline, noradrenaline, dopamine, ephedrine, phenylephrine, argipressin, terlipressin, and desmopressin, were administered at similar frequencies in both groups. In contrast, vitamin K, coagulation factor concentrates in general, and prothrombin complex concentrates specifically were significantly more often administered to patients with GIB under VKA compared with DOACs (all *p*¡0.01, Table 4).

### Outcome

The outcome measures are presented in Table 5. The majority of the patients (95.6% in the DOAC group and 97.0% in the VKA group) recovered fully; four patients (three patients under DOACs and one patient under VKA) recovered with permanent sequelae, and there were five deaths (two in the DOAC group and three in the VKA group). The length of hospitalization did not differ between the groups (median, 9 days in the DOAC group, and 9.75 days in the VKA group; *p* = 0.783). The rebleeding rate during the study period did not differ between the DOAC (16.7%) and VKA groups (25.4%; *p* = 0.131).

**Table 5:** Outcome measures and statistics. Numbers are n (%) unless otherwise stated. Asterisks (*) represent chi-square tests.

	Entire study population (*n* = 248)	DOAC (*n* = 114)	VKA (*n* = 134)	*P*-Value
Length of hospitalization (days), median [IQR]	11 [10]	10 [9]	11.5 [9.75]	0.783
Admission to ICU	59 (23.8)	22 (19.3)	37 (27.6)	0.167*
Rebleeding under OAC during period of study	53 (21.4)	19 (16.7)	34 (25.4)	0.131*
Outcome				
Recovered	239 (96.4)	109 (95.6)	130 (97.0)	
Recovered with permanent sequelae	4 (1.6)	3 (2.6)	1 (0.7)	0.487*
Death	5 (2.0)	2 (1.8)	3 (2.2)	

**Notes.**

DOAC direct oral anticoagulant VKA vitamin K antagonist IQR interquartile range ICU intensive care unit OAC oral anticoagulation

## Discussion

In this retrospective comparison of gastrointestinal bleeding following treatment with DOAC or VKA, we found that patients with VKAs had more comorbidities, were prescribed more potentially interacting medications, and showed higher HAS-BLED bleeding risk scores than patients using DOACs. Polypharmacy was common in both the groups. Pharmacodynamic drug-drug interactions were more common than pharmacokinetic interactions. One-quarter of the patients in the DOAC group did not receive the correct dose (adjusted for renal function, age, weight, and indications). Co-medication with amiodarone appeared to be a risk factor for GIB in both groups. The latency time between starting anticoagulation and developing a GIB was significantly shorter in the DOAC group than in the VKA group. Pharmacological interventions were more common in patients receiving VKAs than DOACs. Nevertheless, no differences were observed in the severity or outcome of GIB.

### Patient characteristics

Generally, patients prescribed VKA had more comorbidities and higher HAS-BLED scores at admission than patients receiving DOACs. Although the European Society of Cardiology (ESC) recommended the use of DOACs in patients with nvAF in 2012^26^, the 2013 “PREFER in AF” registry in seven European countries showed that DOACs were primarily prescribed to younger patients with fewer risk factors^1^, as we also observed. Possible reasons for this include that DOACs were not widely available in many countries at that time, the HAS-BLED score has not been validated for DOACs and a lack of monitoring and antidotes for DOACs meaning that physicians might have elected to manage patients at high bleeding risk with VKAs rather than DOACs^20,27^. However, the use of DOACs has increased over the years^3,28^, as was also observed in the present analysis (Figure 2).

The prescriptions for PPI and antacids prior to hospitalization were equally distributed in both groups. Although prophylactic prescription of PPIs can reduce the incidence of GIBs in high-risk patients receiving DOACs^29^, 95 patients (38.5%) did not receive a gastroprotective drug such as a PPI prior to hospitalization.

### OAC dose

DOAC users frequently received doses that were not adapted according to indication, current renal function, age, weight, and interacting medications (25% of the DOAC group). However, the measured renal function may have represented GIB-induced (pre-renal) renal impairment, not chronic renal impairment. More than half of the VKA-treated patients had supra-therapeutic INR values at admission.

We found that patients receiving DOACs in combination with amiodarone were more often underdosed (21%) than those who were anticoagulated with DOACs who were not receiving amiodarone (7%) or patients anticoagulated with VKAs (no patients had a subtherapeutic INR). The differences were statistically significant. Therefore, despite receiving lower DOAC doses, more patients developed a GIB when concomitantly treated with amiodarone. According to the product information of rivaroxaban, apixaban, edoxaban, and dabigatran, there is no clinically relevant interaction with amiodarone, and the dose of DOACs does not need to be reduced ^30,31,32,33^. Furthermore, the product information for rivaroxaban explicitly indicates that there is no increased risk of bleeding in combination with amiodarone^30^. Our data and more recent data from other large prospective studies refute this^15,34,35^. In our opinion, product information should be updated to warn users about this interaction. Whether DOACs require dose reduction in combination with amiodarone should be investigated in prospective clinical studies.

The median latency time between the start of OAC prescription and GIB was significantly shorter in the DOAC group than in the VKA group. This observation is supported by Shimada et al., who found that 70% of GIBs occurred within the first month of starting DOACs and emphasized the need for exercise vigilance and patient education during this period^36^. The longer latency time until GIB that we observed for the VKA group is similar to the findings of Chen et al., who found that the time between starting a VKA and the first onset of GIB was 41.0 ±  58.4 months (mean ±  SD)^37^.

### GIB severity and management

There was no difference in predicted severity, mortality, or re-bleeding, or in the need for clinical interventions between the two groups, as assessed using the GBS, Rockall, and CURE hemostasis prognosis scores. Nevertheless, patients in the VKA group received more pharmacological interventions than those in the DOAC group. One reason for this might be that a specific antidote for all VKAs was available during the study period, whereas only dabigatran had a specific reversal agent (idarucizumab) in the DOAC group. Andexanet alfa, a recombinant modified human factor Xa protein for the reversal of factor Xa inhibitors, was first approved by the FDA in 2018, after the data collection for this study was completed. Andexanet alfa is effective in reversing rivaroxaban and apixaban anticoagulation because of the reduction in anti-factor Xa activity in healthy patients and those with acute major bleeds^38,39^. Only two of the nine patients taking dabigatran received idaruzicumab. This may be due to the FDA approval of idarucizumab in 2015^40^. Moreover, guidelines recommend administering idaruzicumab only if bleeding is life-threatening and other interventions have been ineffective^41^.

Not only did patients in the VKA group receive more antagonists, but they also received more clotting factors (*p* = 0.003), especially Prothromplex^®^ and Beriplex^®^. Both products consist of factors II, VII, IX, and X, which are vitamin K-dependent coagulation factors. In patients with DOACs only limited data for the use of clotting factor products is available^41^ and the administration of these products is off-label^42,43^.

### Outcome

The vast majority (96.4%) of the patients recovered from GIB, 1.6% recovered with permanent sequelae, and 2% died. Similar results were reported by Mark et al. in a retrospective study of GIB in patients treated with OACs, with a mortality rate of 2.9%^44^.

### Limitations

The important limitations of our study were its retrospective nature, missing information, and small sample size. The latter precludes analysis by treatment indication or specific type of DOAC or VKA. Nevertheless, it is a real-life, single-center study conducted when DOACs were recently licensed.

## Conclusions

In conclusion, this retrospective study shows that patients receiving VKAs seem to have more established risk factors for bleeding those receiving DOACs. They had higher CCI and HAS-BLED scores and received more interacting medications than patients in the DOAC group. Individualizing DOAC therapy in clinical practice is challenging, as shown by the fact that the doses in the DOAC group were often not correctly adapted to the patients’ renal function, age, weight, and indications. Co-medication with amiodarone appeared to be a risk factor for GIB with DOACs, as supported by evidence from other studies. This interaction requires further investigation and attention, as it is currently not rated in the product information as being clinically significant or requiring DOAC dose reduction. Latency was significantly shorter in the DOAC group, indicating that clinicians should be particularly aware of the risk of GIB during the first months after commencing a DOAC and of the higher risk of late bleeding among VKA users.

## Author CRediT statement

Conceptualisation: AL, YS / Data curation: KD, AL, LN / Formal Analysis: KD, AL, LN, YS / Funding Acquisition: None / Investigation: All authors / Methodology: KD, AL, YS / Project Administration: KD, AL, LN, YS / Resources: SB, LD, AH, AL / Software: n/a / Supervision: AL / Validation: All authors / Visualization: LN, YS / Writing-original draft: KD, AL, LN, YS / Writing-review & editing: All authors.

## Supplementary Material

A supplementary file is available with the online version of this article showing the classifications of co-medications.
